# Heart rate acceleration and deceleration capacities associated with circadian blood pressure variation

**DOI:** 10.1111/anec.12748

**Published:** 2020-02-27

**Authors:** Liyuan Yan, Jianling Jin, Xin Zhao, Xingmei Huang, Wei Zhu, Shili Jiang, Meiwen Gao, Jiamin Yuan

**Affiliations:** ^1^ Department of Cardiology the First Affiliated Hospital of Soochow University Suzhou China; ^2^ Department of Electrocardiography the First Affiliated Hospital of Soochow University Suzhou China

**Keywords:** heart rate variability, Holter/event recorders

## Abstract

**Background:**

Heart rate acceleration and deceleration capacities are novel parameters that can quantify sympathetic and vagal modulation. However, how acceleration and deceleration capacities associated with circadian blood pressure (BP) variation remains unknown.

**Methods:**

A total of 141 patients with essential hypertension were included in our study. Based on the nocturnal decline rate of systolic BP (SBP), patients were divided into two groups, as dippers and nondippers. Baseline demographic characteristics, ambulatory blood pressure monitoring (ABPM) parameters, Holter recordings, and echocardiographic parameters were collected.

**Results:**

The absolute values of acceleration capacity (AC) (−7.75 [−8.45 ~ −6.3] ms vs. −6.6 [−8.25 ~ −5.2] ms, *p* = .047) and deceleration capacity (DC) (7.35 [6.1 ~ 8.1] ms vs. 6.3 [5.1 ~ 7.6] ms, *p* = .042) were significantly higher in dippers than in nondippers. By multivariate logistic regression analysis, left atrial diameter (LAd) was found to be an independent risk factor for nondipper status in acceleration capacity model (odds ratio 1.174, 95% confidence interval 1.019–1.354, *p* = .027) and deceleration model (odds ratio 1.146, 95% confidence interval 1.003–1.309, *p* = .045). Sleep SBP was positively correlated to acceleration capacity (*r* = .256, *p* = .002) and negatively correlated to deceleration capacity (*r* = −.194, *p* = .021).

**Conclusions:**

The absolute values of acceleration capacity and deceleration capacity were higher in patients with dipper hypertension than in patients with nondipper hypertension. However, acceleration and deceleration capacities were not independent risk factors for blunted BP variation. Sleep SBP seemed to be better correlated to the impairment of autonomic nervous system (ANS) function than other ABPM parameters.

## 1 INTRODUCTION

Hypertension, which is one of the commonest chronic disease, plays an important role in target organ damage and serves as an independent risk factor for stroke, coronary artery disease, and renal insufficiency (Messerli, Williams, & Ritz, 2007). Recently, international guidelines recommend an early, strict, and all‐day BP control (Whelton et al., 2018; Williams et al., 2018). As a result, out‐of‐office BP measurement, including home blood pressure monitoring and ambulatory blood pressure monitoring (ABPM), is increasingly used to detect nocturnal hypertension, to evaluate the efficacy of antihypertensive drugs and to analyze the BP rhythm (Cho, 2019). ABPM can help to reveal the circadian variation of BP. According to decrease of average systolic BP (SBP) during nighttime than during daytime (nocturnal dip rate), circadian BP variation was divided into dipper (nocturnal dip rate ≥ 10%) and nondipper (nocturnal dip rate <10%) (Pickering, 1990). Dipper is considered to be a normal physiologic status, while nondipper is related to increase risk of target organ damage and cardiovascular mortality (Hermida, Ayala, & Portaluppi, 2007; Ohkubo et al., 2002).

Previous studies have shown that ANS was involved in the control of circadian BP variation (Dauphinot et al., 2010; Kohara, Nishida, Maguchi, & Hiwada, 1995). In their studies, indices of heart rate variability were used to reflect the function of ANS. Heart rate acceleration capacity and deceleration capacity calculated through 24‐hr Holter recordings can quantify sympathetic and vagal modulation and predict cardiovascular and total mortality (Arsenos et al., 2016; Bauer et al., 2006). To the best of our knowledge, there was no study investigating the association of acceleration and deceleration capacities with circadian variation of BP. Therefore, our study was designed to investigate the effect of ANS function quantified by the use of acceleration and deceleration capacities on circadian BP rhythm.

## 2 METHODS

### 2.1 Ethical issues

The single‐center retrospective study was performed in full accordance with the principles outlined in the Declaration of Helsinki, and permission was obtained from the ethics committee of Soochow University.

### 2.2 Study population

Our study retrospectively included a total of 141 hypertensive patients admitted to the Cardiology division at the first Affiliated Hospital of Soochow University who simultaneously underwent 24‐hr ambulatory electrocardiograms, transthoracic echocardiography (TTE), and ABPM from January 1, 2017, to February 1, 2019. Hypertension was defined as casual office SBP ≥ 140 mm Hg and/or diastolic BP (DBP) ≥ 90 mm Hg, ABPM daytime SBP ≥ 135 mm Hg and/or DBP ≥ 85 mm Hg, ABPM nighttime SBP ≥ 120 mm Hg and/or DBP ≥ 70 mm Hg, ABPM 24‐hr SBP ≥ 130 mm Hg and/or DBP ≥ 80 mm Hg, previously diagnosed hypertension, or currently using antihypertensive drugs (Williams et al., 2018).

The patients were excluded if they (a) did not have complete medical records; (b) were with more than 1,000 atrial or ventricular premature beats within a whole day; (c) had a history of secondary hypertension, atrial fibrillation, atrial flutter, atrioventricular block, paced rhythm, sick sinus syndrome, diabetes mellitus, chronic liver disease, renal insufficiency, acute coronary syndrome, electrolyte imbalance, obstructive sleep apnea syndrome, or malignant tumor.

### 2.3 ABPM recordings

All patients included in our study underwent 24‐hr ABPM by an ABPM 6100 device (Welch Allyn Corp.) to record the circadian variation of BP. The right arm of hypertensive patients was selected for the placement of cuff. Patients were instructed to keep their daily routine during the monitoring period and to remain calm and maintain their body position when feeling the inflation of cuff. BP was measured every 15 min from 8:00 a.m. to 11:00 p.m. (daytime BP values). BP was measured every 30 min during the nighttime (11:00 p.m.–8:00 a.m.). Recordings with more than 70% of valid BP measurements were considered to be reliable and included in the final analysis. The means of SBP and DBP were calculated at awake, sleep, and 24 hr for every patient. We defined the BP dipping status as the percentage decline in nocturnal SBP from day to night by the formula: (%) 100 × [1 – (sleep SBP/awake SBP)] (Tanriverdi et al., 2018). Dipper hypertension was defined as ≥10% decrease in SBP measurements, while nondipper hypertension was regarded as <10% decrease in SBP measurements (Pickering, 1990). Then, patients included in our study were divided into two groups, as dipper and nondipper.

### 2.4 Holter recordings

A Holter monitor test was performed in every patient included in our study using a portable electrocardiogram device (BI corp.). HRV was measured using a 24‐hr electrocardiographic Holter system (BI corp.). The following indices related to HRV, including time‐domain indices and frequency‐domain indices, were measured. Among time‐domain indices, we analyzed the full‐course standard deviation of the normal‐to‐normal (NN) intervals (SDNN), the mean of all the 5‐min standard deviations of NN intervals during the all‐day period (SDNN index), the square root of the mean of the sum of the squares of differences between adjacent NN intervals (RMSSD), and the number of pairs of successive NN intervals that differ by more than 50 ms divided by the total number of all NN intervals (pNN50). Frequency‐domain indices included high frequency (HF), low frequency (LF), and very low frequency (VLF). Average heart rate, fastest heart rate, and slowest heart rate calculated by the 24‐hr electrocardiographic Holter system (BI corp.) were also collected for every patient. The heart rate acceleration and deceleration capacities were calculated as previous described (Bauer et al., 2006). Firstly, heartbeat intervals were selected as decelerating anchors when >1.00 but ≤1.05 of the preceding heartbeat interval; heartbeat intervals were selected as accelerating anchors when <1.00 but ≥0.95 of the preceding heartbeat interval. Secondly, the segments of heartbeat intervals around accelerating and decelerating anchors were collected. Thirdly, the above segments were aligned at the accelerating and decelerating anchors and the signals of segments were averaged to obtain the phase‐rectified signal averaging signal X(i). In the end, the following formula was used to quantify acceleration capacity and deceleration capacity: AC/DC = [X(0)+X(1)−X(−1)−X(−2)]/4. To avoid the error of manual measurement, the heart rate deceleration capacity and acceleration capacity were also measured by the Holter system (BI corp.). The values of deceleration capacity are over 0 and the values of acceleration capacity are less than 0. Based on the DC values, patients can be divided into high‐risk (DC ≤ 2.5 ms), intermediate‐risk (2.5 ms < DC ≤ 4.5 ms), and low‐risk (DC > 4.5 ms) patients (Bauer et al., 2006).

### 2.5 Echocardiographic evaluation

All patients included in our study underwent TTE by a Sonos 5500 Ultrasound machine (Philips). The following parameters measured by the M‐mode technique for each patient: LAd (normal range: 27–40 mm), left ventricular end‐diastolic diameter (LVEDd, normal range: 35–56 mm), and left ventricular end‐systolic diameter (LVESd, normal range: 20–40 mm). Simpson's biplane method was used to measure the left ventricular ejection fraction (LVEF, normal value >50%).

### 2.6 Statistical analysis

IBM SPSS for Windows version 25.0 (IBM Corp.) was used to perform statistical analysis. Continuous variables with a normal distribution were presented as mean ± standard deviation (*SD*), while continuous variables with a skewed distribution were presented as median (interquartile range, IQR). Continuous variables were compared using independent sample *t* test or the Mann‐Whitney *U* test. Categorical variables, defined as frequency and percent, were compared using the chi‐square test or Fisher's exact test. To assess the correlation between continuous variables, Pearson's correlation coefficients were calculated. Multivariate logistic regression analysis was applied to determine independent risk factors for nondipper BP pattern. In our study, values of *p* less than .05 were considered to be statistically significant.

## 3 RESULTS

In total, 141 hypertensive patients, including 20 dippers (14.2%) and 121 nondippers (85.8%), were included in our retrospective study. Demographic, antihypertensive treatment, variables of ABPM, cardiac electrophysiological data, and echocardiographic parameters were compared between dippers and nondippers. The baseline demographic and clinical characteristics of dipper and nondipper hypertensive patients were shown in Table 1. Awake SBP (132 [128.5 ~ 138.75] mm Hg vs. 125 [114 ~ 135.5] mm Hg, *p* = .017) and awake DBP (78 [73.25 ~ 85] mm Hg vs. 70 [64 ~ 79] mm Hg, *p* = .010) were significantly lower in patients with nondipper hypertension than in patients with dipper hypertension, while age (51.75 ± 13.70 years vs. 62.37 ± 12.64 years, *p* = .001), sleep SBP (116.5 [106.25 ~ 120] mm Hg vs. 122 [113.5 ~ 133] mm Hg, *p* = .005), and sleep DBP (63 [60 ~ 69.5] mm Hg vs. 70 [63.5 ~ 76.5] mm Hg, *p* = .023) were significantly higher in patients with nondipper hypertension than in patients with dipper hypertension. However, there were no significant differences in other demographic and clinical variables between the two groups including antihypertensive medication. The cardiac electrophysiological indexes of included patients were shown in Table 2. The fastest heart rate (118 ± 16.10 bpm vs. 108.36 ± 15.59 bpm, *p* = .012), SDNN (132.5 [112.25 ~ 160.75] ms vs. 104 [86.5 ~ 131] ms, *p* = .003), LF (311.95 [253.025 ~ 591.15] ms^2^ vs. 225.6 [121.85 ~ 380.1] ms^2^, *p* = .012), and deceleration capacity (7.35 [6.1 ~ 8.1] ms vs. 6.3 [5.1 ~ 7.6] ms, *p* = .042) were significantly higher in patients with dipper hypertension than in patients with nondipper hypertension, while heart rate acceleration capacity (−7.75 [−8.45 ~ −6.3] ms vs. −6.6 [−8.25 ~ −5.2] ms, *p* = .047) was significantly lower in dippers than in nondippers. However, other indexes of cardiac electrophysiology showed no significant differences between dippers and nondippers. When it comes to echocardiographic parameters, only LAd (36.5 [35 ~ 38.20] mm vs. 38.26 [35.5 ~ 41] mm, *p* = .019) showed a significantly difference between dippers and nondippers.

**Table 1:** Baseline demographic and clinical characteristics of dipper and nondipper hypertensive patients

	Dipper (*n* = 20)	Nondipper (*n* = 121)	*p*
Age, years	51.75 ± 13.70	62.37 ± 12.64	**.001**
Sex, males, *n* (%)	10 (50)	51 (42.1)	.511
BMI, kg/m^2^	24.53 ± 4.09	24.80 ± 3.15	.735
Smoking, *n* (%)	3 (15)	17 (14)	1.000
Drinking, *n* (%)	3 (15)	10 (8.3)	.397
Medications, *n* (%)			
ACEI/ARBs	11(55)	70 (57.9)	.811
β‐Blockers	5 (25)	42 (34.7)	.393
CCBs	10 (50)	57 (47.1)	.810
Diuretics	1 (5)	29 (24.0)	.075
24 hr SBP, mm Hg	129 (122.5 ~ 133)	124 (114 ~ 134)	.185
24 hr DBP, mm Hg	74.5 (71 ~ 79.75)	70 (64 ~ 79)	.123
Awake SBP, mm Hg	132 (128.5 ~ 138.75)	125 (114 ~ 135.5)	**.017**
Awake DBP, mm Hg	78 (73.25 ~ 85)	70 (64 ~ 79)	**.010**
Sleep SBP, mm Hg	116.5 (106.25 ~ 120)	122 (113.5 ~ 133)	**.005**
Sleep DBP, mm Hg	63 (60 ~ 69.5)	70 (63.5 ~ 76.5)	**.023**

Note

Normally distributed data are presented as means ± standard deviation (*SD*), skewed data are presented as the median (interquartile range), and categorical data are presented as a number (percentage). Values in bold indicate statistical significance (*p* < .05).

Abbreviations: ACEI, angiotensin‐converting enzyme inhibitor; ARB, angiotensin receptor blocker; BMI, body mass index; CCB, calcium channel blocker; DBP, diastolic blood pressure; SBP, systolic blood pressure.

**Table 2:** Twenty‐four‐hr ambulatory electrocardiographic and echocardiographic variables of dipper and nondipper hypertensive patients

	Dipper (*n* = 20)	Nondipper (*n* = 121)	*p*
Heart rate variability			
Average heart rate, bpm	70.5 (64.25 ~ 77.75)	68 (62 ~ 76)	.692
Slowest heart rate, bpm	49.00 ± 6.46	51.95 ± 7.53	.100
Fastest heart rate, bpm	118 ± 16.10	108.36 ± 15.59	**.012**
SDNN, ms	132.5 (112.25 ~ 160.75)	104 (86.5 ~ 131)	**.003**
SDNN index, ms	47.5 (43 ~ 64)	44 (34 ~ 53)	.073
RMSSD, ms	25.5 (20.25 ~ 31)	23 (18 ~ 30)	.280
PNN50, %	6.5 (1.25 ~ 10)	3 (1 ~ 8)	.352
HF, ms^2^	124.6 (84.325 ~ 268.9)	108.8 (69 ~ 188.55)	.249
LF, ms^2^	311.95 (253.025 ~ 591.15)	225.6 (121.85 ~ 380.1)	**.012**
VLF, ms^2^	1,031.8 (838.05 ~ 1,521)	856.8 (530.65 ~ 1,200.65)	.050
Deceleration and acceleration capacity			
Acceleration capacity, ms	−7.75 (−8.45~−6.3)	−6.6 (−8.25~−5.2)	**.047**
Deceleration capacity, ms	7.35 (6.1 ~ 8.1)	6.3 (5.1 ~ 7.6)	**.042**
Echocardiography			
LAd, mm	36.5 (35 ~ 38.20)	38.26 (35.5 ~ 41)	**.019**
LVEDd, mm	47 (45 ~ 48.03)	48 (44 ~ 51)	.307
LVESd, mm	30.5 (26.5 ~ 32)	31 (28 ~ 33)	.301
LVEF, %	65 (61.25 ~ 70.75)	65 (62 ~ 68)	.774

Note

Normally distributed data are presented as means ± standard deviation (*SD*) and skewed data are presented as the median (interquartile range). Values in bold indicate statistical significance (*p* < .05).

Abbreviations: Bpm, beat per min; HF, high frequency; LAd, left atrial diameter; LF, low frequency; LVEDd, left ventricular end‐diastolic diameter; LVEF, left ventricular ejection fraction; LVESd, left ventricular end‐systolic diameter; PNN50, the mean number of times in full course in which the change in successive normal sinus intervals exceeds 50 ms; RMSSD, root mean square of successive differences; SDNN, standard deviation of NN intervals; VLF, very low frequency.

**Figure 1:**
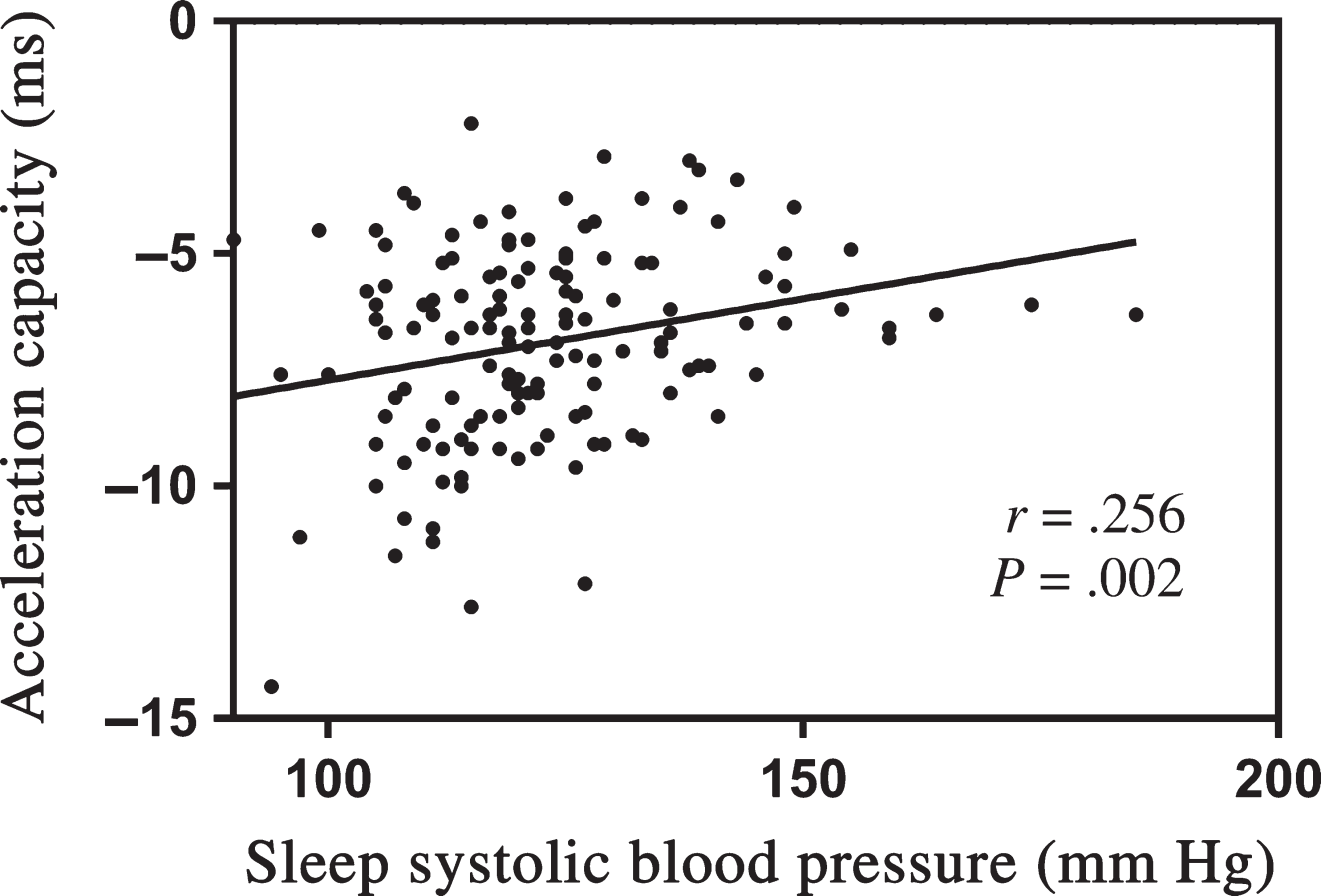
The relationship of acceleration capacity and sleep systolic blood pressure

**Figure 2:**
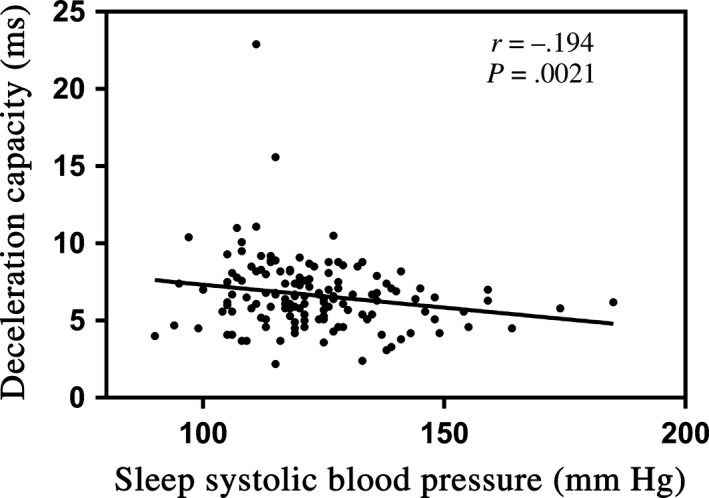
The relationship of deceleration capacity and sleep systolic blood pressure

Correlation analyses among ABPM parameters, echocardiographic variables, heart rate acceleration capacity, and deceleration capacity were shown in Table 3. A weak positive correlation was found between heart rate acceleration capacity and 24 hr SBP (*r* = .194, *p* = .021), and sleep SBP (*r* = .256, *p* = .002; Figure 1). We also found that heart rate acceleration capacity was negatively correlated with nocturnal decline rate of SBP (*r* = −.176, *p* = .037). Age was found to be positively correlated to acceleration capacity (*r* = .349, *p* < .001) and negatively correlated to deceleration capacity (*r* = −.255, *p* = .002). What's more, heart rate deceleration capacity was found to be negatively correlated to sleep SBP (*r* = −.194, *p* = .021; Figure 2). In addition, LAd was the only echocardiographic variable that was correlated to acceleration capacity (*r* = .251, *p* = .003) and deceleration capacity (*r* = −.194, *p* = .021).

**Table 3:** Correlation analysis among ambulatory blood pressure recordings, echocardiographic variables, heart rate acceleration capacity, and deceleration capacity

	AC		DC	
	*r*	*p*	*r*	*p*
Age	.349	**<.001**	−.255	**.002**
24 hr SBP	.194	**.021**	−.162	.055
24 hr DBP	−.060	.483	.033	.694
Awake SBP	.164	.052	−.141	.096
Awake DBP	−.083	.326	.048	.571
Sleep SBP	.256	**.002**	−.194	**.021**
Sleep DBP	.049	.564	−.037	.666
Dip rate	−.176	**.037**	.102	.231
LAd	.251	**.003**	−.194	**.021**
LVEDd	.132	.119	−.152	.072
LVESd	.155	.066	−.140	.099
LVEF	−.161	.056	.099	.243

Note

Values in bold indicate statistical significance (*p* < .05).

Abbrevations: AC, acceleration capacity; DBP, diastolic blood pressure; DC, deceleration capacity; LAd, left atrial diameter; LVEDd, left ventricular end‐diastolic diameter; LVEF, left ventricular ejection fraction; LVESd, left ventricular end‐systolic diameter; SBP, systolic blood pressure.

To determine the independent risk factors of nondipper status, multivariate logistic regression analysis was performed in our study. Age was found to be an independent risk factor for BP nondipper status in both acceleration capacity model (odds ratio 1.069, 95% confidence interval 1.014–1.127, *p* = .013) and deceleration capacity model (odds ratio 1.058, 95% confidence interval 1.007–1.111, *p* = .024; Table 4). LAd was also found to be an independent risk factor for BP nondipper status in acceleration capacity model (odds ratio 1.174, 95% confidence interval 1.019–1.354, *p* = .027) and deceleration capacity model (odds ratio 1.146, 95% confidence interval 1.003–1.309, *p* = .045; Table 4).

**Table 4:** Multivariate logistic regression analysis for circadian BP pattern

	*B*	*SE*	Wald	OR (95%CI)	*p*
Acceleration capacity model					
Acceleration capacity	−.243	.198	1.497	0.784 (0.532–1.157)	.221
Age	.067	.027	6.211	1.069 (1.014–1.127)	**.013**
Fastest heart rate	−.015	.019	0.627	0.985 (0.948–1.023)	.428
SDNN	−.016	.009	2.911	0.984 (0.966–1.002)	.088
LF	−.001	.001	0.412	0.999 (0.997–1.002)	.521
LAd	.161	.073	4.920	1.174 (1.019–1.354)	**.027**
Deceleration capacity model					
Deceleration capacity	.087	.183	0.225	1.091 (0.762–1.560)	.635
Age	.056	.025	5.098	1.058 (1.007–1.111)	**.024**
Fastest heart rate	−.020	.019	1.083	0.980 (0.944–1.018)	.298
SDNN	−.012	.009	1.862	0.988 (0.970–1.005)	.172
LF	<.001	.001	0.115	1.000 (0.997–1.002)	.735
LAd	.136	.068	4.003	1.146 (1.003–1.309)	**.045**

Note

ORs for continuous variables = odds ratio for an increase in 1 unit. Values in bold indicate statistical significance (*p* < .05).

Abbreviations: *B*, logistic coefficient; CI, 95% confidence interval; LAd, left atrial diameter; LF, low frequency; SDNN, standard deviation of RR intervals.

## 4 DISCUSSION

As a normal physiologic state, BP is high during daytime and low during nighttime. Dipper status, which is considered to be a normal physiologic change, is defined as the average sleep SBP decreasing by more than 10% from average awake SBP. Meanwhile, nondipper status demonstrates a blunted nighttime BP drop or even increase in nighttime BP. Previous studies suggested that disturbed circadian BP variation was correlated to damage of target organs and increased cardiovascular morbidity and mortality.

Several factors, including intrinsic factors and extrinsic factors, contribute to disturbed circadian variation of BP. Imbalance of ANS is one of the most important factors for abnormal BP variation (Biaggioni, 2008). Impaired ANS function was found in nondipper hypertensive patients. Kohara et al. (1995) suggested that the function of ANS, evaluated by LF (a Holter index reflecting both sympathetic and vagal activity) and HF (a Holter index reflecting the efferent of vagal activity), was reduced in nondippers. In addition, lower awake LF/HF ratios and asleep/awake ratio for HF were found in nondippers than in dippers, which also suggested reduced ANS activity in hypertensive patients with nondipper status (Kario et al., 1997). Furthermore, a prospective observational cohort showed that a decreased VLF (a Holter index reflecting the efferent sympathetic activity) contributed to the increased risk of shift to BP nondipper status (Dauphinot et al., 2010). However, these parameters of HRV measured in these previous studies cannot adequately quantify autonomic dynamics. Heart rate acceleration and deceleration capacities, as novel indicators of ANS function, are able to quantify sympathetic and vagal modulation (Bauer et al., 2006). Decrease in deceleration capacity was found to be an independent predictor of mortality in postmyocardial infarction patients and an independent predictor of sudden cardiac death in heart failure patients (Arsenos et al., 2016; Bauer et al., 2006). Acceleration capacity was shown to be not only an independent risk factor for dilated cardiomyopathy (DCM), but also a predictor for heart failure exacerbation in DCM patients (Zou et al., 2016). However, to the best of our knowledge, there have been no studies investigating heart rate acceleration and deceleration capacities in hypertensive patients with disturbed BP variation.

In the present study, shortened absolute values of acceleration capacity and deceleration capacity were found in hypertensive patients with nondipper status, which may suggest that decrease in both sympathetic and vagal activity in nondippers with compared to dippers. However, in the multivariate logistic regression analysis, heart rate acceleration capacity and deceleration capacity were not independent risk factors for nondipper hypertension, which may result from the limited number of patients included in our study.

Left atrial diameter was significantly higher in nondippers than in dippers in our study and shown to be an independent risk factor for nondipper status, which were consistent with a previous study focusing on the relationship between echocardiographic parameters and BP variation (Tigen et al., 2009). LAd was also positively correlated to acceleration capacity and negatively correlated to deceleration capacity, which means the increase of LAd may result in impairment of both sympathetic and vagal activity. Enlargement of left atrium may play an important role in autonomic cardiac regulation, because the intrinsic cardiac nerves are mainly located in atrium (Chen, Chen, Fishbein, Lin, & Nattel, 2014).

Age was significantly lower in dipper than in nondippers and an independent risk factor for abnormal BP variation. What's more, age was positively correlated to acceleration capacity and negatively correlated to deceleration capacity. With the change of age, several components of ANS changed, including baroreceptor reflex function, the plasma level of norepinephrine, 6‐adrenoceptor sensitivity, and postreceptor signaling change (Goldstein et al., 1983; Kusiak & Pitha, 1983; Lake, Ziegler, Coleman, & Kopin, 1977; Shimada, Kitazumi, Sadakane, Ogura, & Ozawa, 1985; Vestal, Wood, & Shand, 1979). Thus, the age‐related change of ANS function may result in an increase in blunted nocturnal drop of blood pressure and impairment of ANS activity.

The fastest heart rate was significantly higher in dippers than nondippers. As described above, evaluated by heart rate acceleration capacity and deceleration capacity, nondippers showed impaired sympathetic and vagal activity. The decrease of fastest heart rate in nondippers compared to dippers may result from reduced sympathetic activity (Shaffer, McCraty, & Zerr, 2014). Dippers had significantly higher LF than nondippers in our present study, which was also found in a previous study (Kohara et al., 1995). However, LF was a controversial parameter. Some considered it to be a marker of sympathetic modulation, while others considered it as a parameter reflecting both sympathetic and vagal activity (Heart rate variability. Standards of measurement, physiological interpretation, & clinical use. Task Force of the European Society of Cardiology & the North American Society of Pacing & Electrophysiology", ; Shaffer et al., 2014). SDNN was significantly higher in hypertensive patients with dipper status than in patients with nondipper hypertension. SDNN is a Holter index reflecting the balance between sympathetic and vagal nervous system in heart rate control. Nondippers with lower SDNN indicated the impairment of the sympathovagal balance in patients with abnormal BP variation.

When it comes to the relationship between ABPM parameters and acceleration capacity, and deceleration capacity, acceleration capacity was positively correlated to 24 hr SBP and sleep SBP, and negatively correlated to dip rate. Sleep SBP was the only ABPM parameter that found to be correlated to deceleration capacity. To our surprise, nocturnal decline rate of SBP was not significantly correlated to deceleration capacity. In our present study, sleep SBP seemed to be better correlated to acceleration capacity and deceleration capacity than other ABPM parameters, which means sleep SBP may play a more important role in the change of ANS function than other ABPM parameters. With the increase of sleep SBP, the absolute values of acceleration and deceleration capacity decreased. There have been more and more studies showing nocturnal blood pressure to be a predictor for target organ damage and adverse cardiovascular events (Hansen et al., 2011; Sega et al., 2005). The findings of our study may help to explain previous studies.

The limitations of the present study were as follows. Firstly, there were only 141 patients with hypertension included in our study, which may be one of the reasons for why acceleration capacity and deceleration capacity were not independent risk factors for nondipping status after multivariate logistic regression analyses. Secondly, our study was a retrospective study. More prospective studies are needed to investigate the association of deceleration and acceleration capacities with BP variation.

## 5 CONCLUSIONS

The absolute values of acceleration capacity and deceleration capacity were decreased in patients with nondipper hypertension compared to patients with dipper hypertension. However, acceleration capacity and deceleration capacity were not independent risk factors for BP nondipper status. Age and LAd were found to be independent risk factors for BP nondipper status. Sleep SBP may be better correlated to the impairment of ANS activity than other ABPM parameters.

## CONFLICT OF INTEREST

None declared.

## AUTHOR CONTRIBUTIONS

All authors contributed significantly to this study. Liyuan Yan and Jianling Jin contributed equally to this work and should be considered as co‐first authors. Jiamin Yuan, Liyuan Yan and Jianling Jin designed the study. Xin Zhao and Xingmei Huang collected the data. Wei Zhu and Shili Jiang analyzed the data. Meiwen Gao made the Tables and Figures. Jiamin Yuan, Liyuan Yan and Jianling Jin wrote the paper. All authors reviewed and approved the manuscript.

## ETHICAL APPROVAL

The single‐center retrospective study was performed in full accordance with the principles outlined in the Declaration of Helsinki, and permission was obtained from the ethics committee of Soochow University.
